# Relation of Changes in PEF and FEV_1_ During Salbutamol-Induced Bronchodilation After Methacholine Challenge Test

**DOI:** 10.1155/pm/7675935

**Published:** 2025-07-07

**Authors:** Leon L. Csonka, Antti Tikkakoski, Liisa Vuotari, Jussi Karjalainen, Lauri Lehtimäki

**Affiliations:** ^1^Faculty of Medicine and Health Technology, Tampere University, Tampere, Finland; ^2^Department of Clinical Physiology and Nuclear Medicine, Tampere University Hospital, Tampere, Finland; ^3^Allergy Centre, Tampere University Hospital, Tampere, Finland

**Keywords:** asthma, bronchodilation, diagnostics, home monitoring, methacholine, spirometry

## Abstract

Asthma diagnosis can be confirmed by observing significant bronchodilator response (BDR) through peak expiratory flow (PEF) at home or forced expiratory volume in 1 s (FEV_1_) via spirometry in a clinical setting. We aimed to use the administration of salbutamol after a methacholine challenge test as a model of bronchodilation to study how accurately the change in PEF predicts improvement in lung function, as defined by an increase in FEV_1_. We analyzed 869 adult patients who were administered salbutamol after a methacholine challenge. To compare relative changes in PEF and FEV_1_ during bronchodilation, we used regression analysis and constructed a Bland and Altman plot. ROC analysis, sensitivity, specificity, positive and negative predictive values, and kappa coefficient assessed how precisely increases in PEF detected a 12% and 0.2-L improvement in FEV_1_. The average relative increase in FEV_1_ was significantly greater than that in PEF. The area under the curve in the ROC analysis was 0.844 for PEF change to detect a 12% and 0.2-L increase in FEV_1_. The kappa values for changes in PEF and FEV_1_ ranged from fair to moderate. BDR detected by the recommended 15% and 60 L/min cut-off for PEF identified less than half of true positives, while a 10% cut-off correctly identified close to 75% of them. PEF increase is not a reliable measure of BDR in comparison to FEV_1_ increase, and a 10% improvement in PEF was the least inaccurate cut-off. Substituting the PEF meter with a handheld spirometer should be further investigated for asthma home monitoring.

## 1. Introduction

Asthma stands as one of the most common chronic diseases, impacting almost 340 million individuals worldwide [1]. Asthma typically presents with breathlessness, cough, wheeze, and chest tightness, along with variable expiratory airflow limitation relieved by bronchodilators [2]. The diagnostic framework for asthma remains devoid of universal standardization, resulting in the utilization of a diverse array of different tests. Diagnosing asthma may encompass evaluating bronchodilator response (BDR) using spirometry, monitoring daily fluctuations in peak expiratory flow (PEF), or identifying bronchial hyperreactivity through diverse challenge tests [2, 3].

Following the adoption of objective lung function measures such as home PEF monitoring as a standard practice for diagnosing asthma in Finnish primary care, diagnostic precision saw a marked improvement, alongside positive shifts in various metrics reflecting the national asthma burden [4]. While home PEF monitoring has been well recognized for its utility in asthma diagnosis and management over the years [5], the European Respiratory Society (ERS) takes a nuanced stance. It refrains from promoting PEF monitoring as the primary or solitary diagnostic method. Instead, it emphasizes PEF monitoring as an adjunctive measure, particularly in cases where spirometry outcomes appear normal, especially if access to bronchial challenge testing is not available [3].

Various studies have indicated the limited reliability of PEF as a single reading for assessing lung function during obstruction [6–10]. However, the affordability and simplicity of handheld PEF meters have facilitated home monitoring. This enables the observation of bronchial obstruction and dilation over time. The usefulness of PEF in monitoring asthma is better proved when multiple daily values are recorded over time [9]. While PEF monitoring proves useful in this setting, some studies indicate potential unreliability, even within the home monitoring framework [11]. Different PEF meters are known to yield noncomparable results [12–14]. Furthermore, the method chosen for calculating PEF variability significantly impacts the sensitivity and specificity of PEF monitoring [15]. Accurately assessing the technical reliability of home PEF measurements is challenging, and unreliable measurements due to inadequate technique can consequently compromise the reliability of home PEF monitoring.

For adults, within the framework of home PEF monitoring, the Finnish national asthma guideline defines a significant BDR as PEF increasing at least 15% and 60 L/min following the administration of bronchodilators. To establish a diagnosis of asthma, BDR must occur at least three times during the monitoring period. GINA recommends a 20% improvement in PEF as the threshold for in-clinic BDR testing as an alternative to FEV_1_, but it currently does not recommend BDR testing in the context of home PEF monitoring. According to the Finnish national asthma guideline, ERS asthma guideline, and GINA, during a bronchodilation test conducted with a spirometer, BDR is defined as an increase of at least 12% and 0.2 L in FEV_1_ [2, 3, 16]. GINA and the Finnish national guideline also recommend an increase of at least 12% and 0.2 L in forced vital capacity (FVC) as an alternative definition for BDR, with a 15% and 0.4-L increase providing greater confidence [2, 16]. The joint American Thoracic Society (ATS) and ERS spirometry technical standard guideline recommends using a cut-off value of at least 10% increase in the predicted value for FEV_1_ or FVC [17].

The little existing research investigating the relation between changes in PEF and FEV_1_ during bronchodilation suggests that the accuracy of PEF change in detecting BDR is questionable [18–21]. Additionally, no study directly compared the recommended BDR cut-off values of 15% and 60-L/min PEF increase and 12% and 0.2-L FEV_1_ improvement. Recently, the availability of compact and reliable handheld spirometers tailored for home monitoring has increased, but the applicability of home spirometry monitoring with bronchodilation testing in asthma diagnostics is not well studied yet. Thus, our goal was to utilize salbutamol administration after a methacholine challenge test as a bronchodilation model to study how accurately PEF changes represent airway relaxation, as defined by an improvement in FEV_1_.

## 2. Methods

### 2.1. Study Design

We gathered all methacholine challenge tests with spirometry performed at Tampere University Hospital between November 19, 2018, and April 14, 2021. The subjects had been sent for a methacholine challenge test due to suspected asthma. The exclusion criteria were the standard contraindications for the methacholine challenge test [22]. Additionally, all patients under 18 years of age and those with technically unreliable measurements were excluded from the study. Patient medical history was collected from records. As this was a retrospective study, ethical approval was not required.

### 2.2. Spirometry and Methacholine Challenge Protocol

Spirometry was performed according to international guidelines [23], using reference values for Finnish adults [24]. The patients' blowing technique, along with the technical reliability and reproducibility of the measurements, was visually evaluated by trained physicians in accordance with international guidelines [25]. Reliability of measurements was established if all spirometry blows during the methacholine challenge met the quality standards outlined in international guidelines [25]. A Vyntus Pneumo spirometer and Carefusion Vyntus APS dosimeter (Vyaire Medical, Illinois, United States) were used to perform the methacholine challenge, in accordance with ERS recommendations [26]. Experienced technicians conducted the methacholine challenge. Following the first spirometry measurements, a saline solution was administered as a diluent step. Subsequently, methacholine doses of 18, 72, 270, 810, and 2600 *μ*g were given at 5-min intervals. After each methacholine dose, spirometry measurements were repeated using the dosimetric method by Nieminen et al. [27] according to Finnish guidelines [22]. Following the challenge, bronchodilator medication (salbutamol 400 *μ*g) was administered using a pMDI and a spacer (Ventoline Evohaler and Volumatic, GSK), and a final spirometry measurement was recorded.

### 2.3. Statistics

Data analysis was performed using IBM SPSS Statistics for Windows, Version 27 (IBM Corp, New York, United States). Microsoft Excel for Microsoft 365 MSO (Version 2110 Build 16.0.14527.20234) (Washington, United States) was used for additional calculations. The relative decrease in FEV_1_ and PEF after methacholine was calculated as follows: ([(premethacholine FEV_1_ or PEF) − (postmethacholine FEV_1_ or PEF)]/(premethacholine FEV1 or PEF))∗100. The relative increase in FEV_1_ and PEF after bronchodilation was calculated as follows: ([(postbronchodilator FEV_1_ or PEF) − (prebronchodilator FEV_1_ or PEF)]/(prebronchodilator FEV_1_ or PEF))∗100. A regression analysis was carried out to examine the correlation between relative changes in FEV_1_ and PEF during bronchodilation. A Bland–Altman plot was used to illustrate the differences in relative changes between FEV_1_ and PEF [28, 29]. Receiver operating characteristic (ROC), sensitivity ([true positive/(true positive + false negative)] × 100), specificity ([true negative/(false positive + true negative)] × 100), PPV ([true positive/(true positive + false positive)] × 100), NPV ([true negative/(false negative + true negative)] × 100), and Cohen's kappa coefficient were employed to compare the increases in FEV_1_ and PEF as outcome measures. An additional analysis based on the ATS/ERS technical spirometry standard recommendation to use an increase in FEV_1_ of at least 10% of predicted value as the definition for BDR was performed as a supplementary analysis [17]. Increase in FEV_1_ % predicted was calculated as ([(postbronchodilator FEV_1_) − (prebronchodilator FEV_1_)]/(predicted FEV_1_))∗100. Increase in PEF % predicted was calculated as follows: ([(postbronchodilator PEF) − (prebronchodilator PEF)]/(predicted PEF))∗100 (Supporting Information). To assess whether the relationship between changes in PEF and FEV_1_ is influenced by age, sex, prebronchodilator lung function, or degree of response to methacholine, we divided the population by sex, median age, and median prebronchodilator FEV_1_ % predicted, as well as into groups who experienced either at least a 20% decrease or less than a 20% decrease in FEV_1_ following methacholine inhalation. We then analyzed whether the differences in sensitivity, specificity, PPV, and NPV between these subgroups were significant using a two-sample test for equality of proportions.

## 3. Results

Throughout the study duration, 971 adults participated in a methacholine challenge test. After excluding 102 tests due to technical unreliability, 869 patients with reliable measurements remained. The average age of the participants was 47 years. One-third of the participants were male, and more than one-third had a history of smoking or were active smokers. On average, the patients had normal lung function prior to the methacholine challenge. After the highest dose of methacholine was administered, lung function significantly worsened, and the relative decrease was slightly larger in FEV_1_ compared to PEF. After salbutamol inhalation, almost half of the patients experienced BDR, as defined by a 12% and 0.2-L increase in FEV_1_. Contingent upon the cut-off value, a significant PEF increase was observed in 17.1%–47.4% of the participants. Using the Finnish recommended 15% and 60-L/min cut-off value, 27.5% of patients were classified as having BDR, while the GINA recommended 20% increase in PEF categorized 24.2% of patients as having BDR (Table 1).

**Figure 1:**
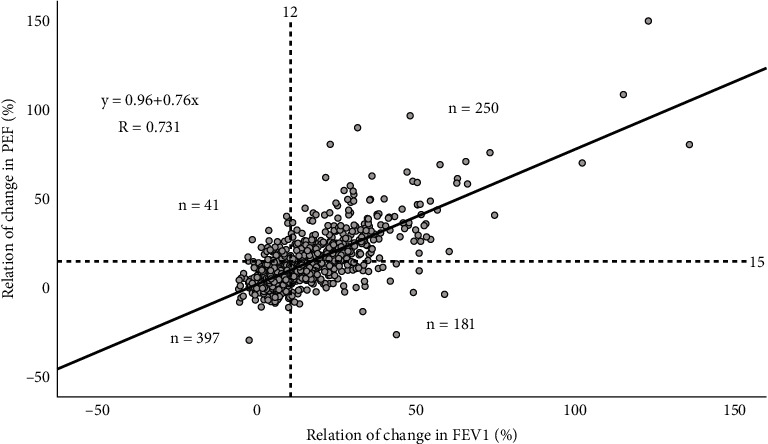
Relative change in PEF in relation to relative change in FEV_1_ during bronchodilation. Horizontal and vertical dotted lines represent increases of 15% and 12% in PEF and FEV_1_, respectively.

Figure 1 presents the relation between relative change in FEV_1_ and relative change in PEF during bronchodilation. A strong positive linear relationship was observed between changes in FEV_1_ and PEF. The formula of the correlation line was *y* = 0.96 + 0.76*x*, meaning the relative change in FEV_1_ was, on average, more than the relative change in PEF after a 4% increase in FEV_1_. The correlation coefficient was 0.731. Of the 869 patients examined, 397 did not meet the criteria for either a 12% increase in FEV_1_ or a 15% improvement in PEF. Moreover, 250 patients achieved both criteria, while 41 attained a 15% increase in PEF without a corresponding 12% improvement in FEV_1_, and 181 achieved a 12% increase in FEV_1_ without a 15% improvement in PEF.

**Figure 2:**
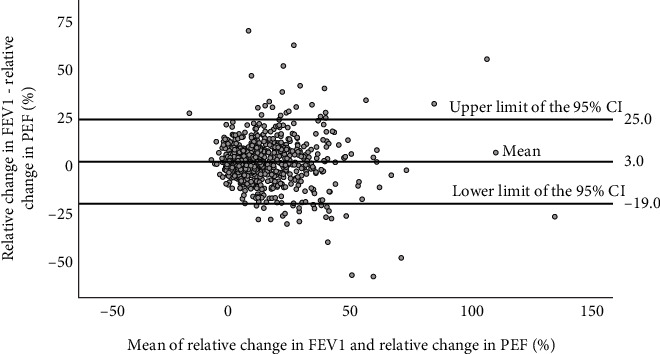
Bland and Altman plot of relative change in PEF and FEV_1_ during bronchodilation. While the mean difference between changes in PEF and FEV_1_ only reached 3.0%, the 95% confidence interval (CI) ranged from −19.0% to 25.0%.

Figure 2 presents a Bland and Altman plot of the relative changes in FEV_1_ and PEF during bronchodilation. The mean signed difference between changes in FEV_1_ and PEF was 3.0%, indicating a larger average increase in FEV_1_ compared to PEF. This concurs with the findings from Figure 1, showing that the relative FEV_1_ increase was mostly greater than the relative PEF improvement. The 95% confidence interval (CI) for the difference between changes in FEV_1_ and PEF was large and varied from −19.0% to 25.0%. The majority of study participants showed some degree of improvement in lung function following salbutamol administration. Most data points reflect this shift to the right on the *x*-axis, signifying some amount of bronchodilation.

**Figure 3:**
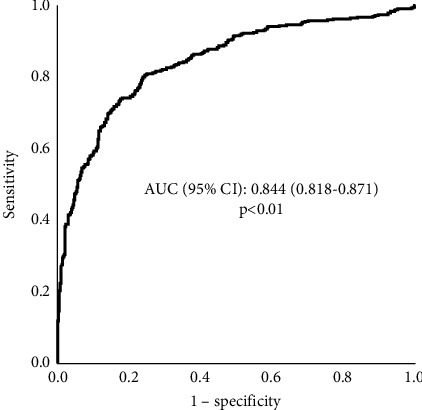
ROC analysis illustrating the association between PEF change and a 12% and 0.2-L increase in FEV_1_ during bronchodilation.

Figure 3 shows a ROC curve comparing changes in PEF with a 12% and 0.2-L increase in FEV_1_ during bronchodilation. The area under the curve (AUC) was 0.844, and the 95% CI ranged from 0.818 to 0.871 (*p* < 0.01). According to the AUC, the ability of PEF change to predict a 12% and 0.2-L increase in FEV_1_ was classified as good [30].

Table 2 shows the sensitivity, specificity, PPV, NPV, and Cohen's kappa coefficient for various PEF increase cut-offs in detecting a 12% and 0.2-L improvement in FEV_1_ after salbutamol inhalation. Lower cut-off values were associated with higher sensitivity and NPV in detecting a 12% and 0.2-L improvement in FEV_1_. In comparison, higher cut-off values achieved improved specificity and PPV. Cohen's kappa ranged between fair and moderate, with values between 0.313 and 0.541 [31]. Judging by kappa value, 10% seemed to be the most accurate PEF cut-off for detecting a 12% and 0.2-L improvement in FEV_1_.

We performed additional analyses based on the ATS/ERS technical spirometry standard recommendation to use an increase in FEV_1_ of at least 10% of predicted value as the definition for BDR [17]. The results of this analysis were similar to those using a BDR definition based on change in relation to baseline, as presented in Table 2, with no critical differences, except that PEF was slightly less accurate when BDR was defined in relation to predicted values (Tables S1 and S2).

We found that after splitting the population based on the prebronchodilator FEV_1_ median value (80% of predicted), median age (47 years), or sex, there were no marked differences in the relation between PEF and FEV_1_ changes across the groups in these subanalyses. We also compared those who experienced a reduction of at least 20% in FEV_1_ after methacholine inhalation, and those who experienced less than a 20% decrease, and found that PEF increase was less accurate at correctly identifying bronchodilation in patients with more severe obstruction before bronchodilation. The different PEF cut-off values were ranked similarly by accuracy across both groups, with a 10% improvement in PEF being the least inaccurate, based on the kappa coefficient (data not shown).

## 4. Discussion

We found that PEF increase was not accurate at detecting bronchodilation as defined by an improvement in FEV_1_. Increase in PEF had especially poor accuracy in patients with more severe obstruction prior to bronchodilation. While the correlation between relative change in FEV_1_ and relative change in PEF was strong, FEV_1_ increased significantly more after bronchodilation. We found increase in PEF to have better specificity and PPV than sensitivity and NPV. The two different cut-off values recommended by the Finnish national asthma guideline and GINA achieved similar results in terms of diagnostic accuracy. Both currently recommended cut-off values identified under half of the true positives based on FEV_1_ improvement. Using the same PEF cut-offs, over one-third of the negative results were false in both cases [2, 16]. Even when using the 10% cut-off for PEF increase, which yielded the best kappa value, the diagnostic characteristics of PEF change remained relatively poor. Although our ROC analysis achieved a good AUC, there was a significant difference in the population detected based on increase in PEF compared to the group selected using improvement in FEV_1_.

Dekker et al. studied the ability of an absolute increase in PEF of 60 L/min in bronchodilation test to predict either a 9% improvement in FEV_1_ percent predicted or an absolute increase in FEV_1_ of 0.19 L. Their findings aligned with ours, showing that the specificity and PPV of PEF change were significantly higher than the sensitivity and NPV for detecting increases in FEV_1_. Although they used the recommended 400 *μ*g of salbutamol, they recorded FEV_1_ and PEF on separate devices, which could introduce variability. Their study population was relatively small, with 73 participants, and had a significantly higher average age of 62 years compared to ours. Notably, they also included only patients with diagnoses of asthma or COPD. These factors, along with the significant differences in cut-off values and calculation methods, may lead to variation in the results [19].

Aggarwal et al. investigated the accuracy of various increases in PEF in detecting a 12% improvement in FEV_1_. Their findings were similar to ours, with one notable difference: PEF consistently achieved a higher NPV than PPV in their study, which contrasted with our results. They also found that the sensitivity of the recommended PEF increase of 15% and 60-L/min was very poor, while the specificity was better. Overall, their results indicated slightly worse diagnostic characteristics for PEF compared to ours, except for NPV. Aggarwal et al.'s study had a large population that well-represented the general population, and they used the recommended dose of salbutamol. However, their definition of BDR did not include the recommended absolute FEV_1_ increase of 0.2 L. Additionally, they recorded FEV_1_ and PEF on different devices, which could affect the results [32].

Thiadens et al. studied the effectiveness of various PEF increases in predicting a 12% and 0.2-L improvement in FEV_1_. Their findings largely aligned with ours, but similar to Aggarwal et al., they found that the NPV of PEF increase was higher than the PPV. Sensitivities and specificities were comparable across most cut-off values; however, at higher cut-offs, sensitivity declined more significantly in their results. For a 15% increase in PEF, they reported a sensitivity of 42% and a specificity of 93%, closely matching our results. However, their PPV was 37%, significantly lower than our 85%, while their NPV was 93%, compared to our 69% for detecting a 12% and 0.2-L improvement in FEV_1_. This difference may stem from the fact that PPV and NPV, unlike sensitivity and specificity, are influenced by the prevalence of the condition the test is designed to detect. In contrast to our study, Thiadens et al. did not induce airway obstruction before administering bronchodilators, and they excluded all patients with COPD or asthma, a population known to show stronger BDRs. These factors resulted in a BDR prevalence manyfold lower than in our sample, likely contributing to the observed differences in PPV and NPV. They administered the recommended dose of salbutamol and measured FEV_1_ and PEF using the same device and expiratory maneuver, minimizing potential result skewing. While they used the recommended cut-off value for FEV_1_, they did not evaluate PEF cut-off values that combine relative and absolute changes, such as the Finnish recommended 15% and 60 L/min. Relative change in lung function was also calculated using percent predicted values instead of comparing prebronchodilator measurements to postbronchodilator values [21].

Ozturk et al. examined the ability of two relative PEF increases, 15% and 20%, to detect a 12% improvement in FEV_1_, dividing the participants into two age groups: under and over 60 years. The diagnostic accuracy of PEF change varied significantly with age and the chosen cut-off value, resulting in conflicting outcomes between groups. Those results also largely contradicted our findings. In patients over 60, sensitivity, specificity, and PPV were lower compared to those under 60, while NPV was slightly better. For a 15% PEF cut-off, sensitivity exceeded specificity in both age groups. PPV was higher than NPV in subjects under 60, whereas NPV was higher in those over 60. Although they used the recommended dose of salbutamol, FEV_1_ and PEF were measured with different equipment. The relatively small population, further divided into smaller groups, may account for some discrepancies with our results. Additionally, they excluded individuals with asthma and COPD and did not include absolute values alongside the relative change in their cut-off values for FEV_1_ and PEF [20].

Home PEF monitoring has been researched extensively and is commonly utilized and recommended. In contrast, home spirometry is neither widely practiced nor recommended [2, 3, 16]. In Finland, home PEF monitoring involves measurements twice daily for 2 weeks, both before and after inhalation of a bronchodilator, typically salbutamol. Asthma can be diagnosed if BDR is observed at least thrice during this period [16]. Internationally, however, home PEF monitoring typically focuses on measuring natural diurnal PEF variability alone, without assessing BDR [2, 3]. It is known that the sensitivity and specificity of PEF change are less than ideal in identifying bronchoconstriction, which is another method for diagnosing asthma during home PEF monitoring [6, 7].

Monitoring PEF with a PEF meter rather than a spirometer complicates quality control. While consistent PEF values are required, spirometry allows for visual assessment of flow volume curves, identifying potential technical issues during exhalation. Our study excluded unreliable measurements, but this would be nearly impossible in a home setting with only PEF values. Consequently, increase in PEF may be an even less reliable indicator of BDR during home monitoring. We have also studied the relationship between changes in PEF and FEV_1_ in children during bronchodilation after an exercise challenge, and those findings further indicate that PEF is inaccurate in detecting BDR (in review). However, because children's airways are smaller, less rigid, and more prone to obstruction, the relationship between changes in FEV_1_ and PEF during bronchodilation may differ from adults. Even in adults, the relationship between changes in FEV_1_ and PEF seems to behave differently depending on age, and it is known that respiratory muscle strength and PEF decline with age [20].

The factors discussed above call into question the future of PEF meters in home monitoring. Since change in PEF seems to reflect lung function poorly in other settings, spirometry could also be more accurate for home settings. Replacing PEF meters with handheld microspirometers should be considered. PEF meters are generally more affordable than microspirometers, providing a cost advantage. However, the increased diagnostic accuracy of microspirometers may enhance resource allocation, potentially offsetting the initial cost difference over time.

Findings on the accuracy of handheld spirometers are mixed, with the chosen manufacturer influencing results. Some studies suggest that portable microspirometers provide accurate results comparable to gold-standard laboratory spirometers [33–35], while others indicate that their results may not be interchangeable with traditional laboratory spirometry [36, 37]. Additionally, studies on the feasibility of home spirometry monitoring have yielded conflicting results. Some found it highly efficacious, useful, and well-received with high compliance [38–40]. However, other research indicated that home spirometry is inconsistent and of lower quality than spirometry performed in supervised clinical settings or that its usefulness in the asthma diagnostic process is limited [41, 42]. While home spirometry shows promise for remote monitoring of lung function, its effectiveness remains unclear, highlighting the need for further research, particularly in evaluating its clinical usefulness for diagnosing asthma.

Our study was performed at one hospital, and there could be variation in the results if the analysis was based on samples from other medical centers employing different bronchodilation protocols or equipment. While smoking data were unavailable for some patients, there is no evidence to suggest that the fraction of smokers differed significantly among those without this data. Additionally, it is essential to note that not all participants in our study had asthma, which could introduce variability in the relationship between changes in PEF and FEV_1_ among individuals with and without asthma. In our population, obstruction was directly induced prior to bronchodilation. While this approach could be considered a limitation, as induced obstruction may behave differently during bronchodilation compared to naturally occurring obstruction associated with exercise, allergens, or infections, it also offers distinct advantages. Specifically, studying a population with induced obstruction allowed us to achieve a high proportion of fairly marked bronchodilator effects and thereby a higher signal-to-noise ratio.

Typically, a diagnosis of asthma based on BDR is not established during methacholine challenge testing [2, 3, 16]. However, our aim was not to evaluate the clinical value of PEF change in this context but rather to study the technical relationship between increase in PEF and improvement in FEV_1_ during bronchodilation. It should be noted that while we did utilize the recommended cut-off value of 12% and 0.2-L increase in FEV_1_, the evidential basis for this recommendation lacks strong support. Current research is insufficient to thoroughly assess the sensitivity of different FEV_1_ cut-off values for BDR in distinguishing patients with asthma from healthy individuals [43]. PEF is believed to primarily indicate the level of dilation in the large airways, whereas FEV_1_ is thought to represent both small and large airways [9]. This distinction may help explain some of the variations observed in this investigation. Nevertheless, there is a lack of experimental data on the specific airway regions to which PEF and FEV_1_ and their changes correspond.

Our study had several notable strengths. We had a large sample of 869 subjects and a meticulous selection process whereby each measurement was individually analyzed for technical reliability. We excluded all unreliable measurements, which enabled us to more accurately examine the relationship between changes in PEF and FEV_1_. Our findings are relevant to the general adult population, as the age distribution of participants in our study was broad and children were excluded. We performed additional analyses using an alternative definition of BDR, and the similarity of these results to our main analyses further confirmed our findings and increased confidence in their validity [17]. Using a PEF meter for PEF measurements might have yielded different results due to the variation between device types [44]. Our previous findings showed that using the same blowing technique with either a laboratory spirometer or a PEF meter resulted in small but statistically significant differences in PEF outcomes. However, further analysis revealed that these differences were not clinically relevant (unpublished).

Blowing technique may also vary depending on the device type. In typical PEF recordings, the exhalation is shorter than the longer blow required for spirometry. Some studies indicate that a prolonged, spirometry-style expiratory maneuver may produce lower PEF values compared to the brief, explosive technique associated with PEF meters. The magnitude of this difference varies, with some studies reporting significant discrepancies while others find minimal differences [45, 46]. Our previous investigation demonstrated that both short and long expiratory efforts yield clinically similar PEF measurements, regardless of whether a PEF meter or a spirometer is used [7]. This highlights that PEF measurements are likely consistent enough from a clinical perspective, whether recorded with the spirometer used in our study or a PEF meter. However, variations may exist among different types of PEF meters, and our evaluation was limited to one type [12, 14].

In conclusion, we demonstrated in relation to salbutamol administration after a methacholine challenge test that the increase in PEF after bronchodilation is not an accurate method of measuring improvement in lung function, as defined by the increase in FEV_1_. PEF increase appears to have greater specificity than sensitivity, with higher PPV compared to NPV. The recommended cut-off for PEF increase, 15% and 60 L/min, has poor sensitivity for detecting improvement in FEV_1_, while a 10% cut-off value was found to be better overall. Incorporating PEF monitoring in asthma diagnostics and management allows for the detection of variability and improvement in lung function. However, it is essential to acknowledge that PEF increase may not reliably indicate reversibility of airway obstruction. Replacing the PEF meter with a portable spirometer could enhance the accuracy of asthma home monitoring and should thus be investigated further.

## Figures and Tables

**Table 1:** Characteristics of 869 study subjects with technically reliable measurements in the methacholine challenge and BDR tests.

**Characteristics**	**Value**
Age (years)	47.4 (16.4)
Gender (*n*)	
Female	581 (66.9)
Male	288 (33.1)
Height (cm)	169.2 (9.4)
Weight (kg)	80.9 (18.4)
BMI (kg/m^2^)	28.2 (6.1)
Smoking status (*n*)	
Data not available	162 (18.6)
Current smoker	56 (6.4)
Ex-smoker	207 (23.8)
Never smoker	444 (51.1)
Lung function at baseline (% predicted)	
FVC	96.2 (13.1)
FEV_1_	93.7 (13.6)
FEV_1_/FVC	97.4 (8.0)
PEF	91.5 (21.0)
FEV_1_ after last dose of methacholine (% predicted)	80.1 (16.6)
PEF after last dose of methacholine (% predicted)	82.0 (16.8)
Relative decrease in FEV_1_ after last dose of methacholine (%)	14.9 (9.6)
Relative decrease in PEF after last dose of methacholine (%)	13.2 (9.7)
Subjects with a ≥ 20% decrease in FEV_1_ after last dose of methacholine (*n*)	265 (30.5)
Bronchodilator response (*n*)	
≥ 12% improvement in FEV_1_	431 (49.6)
≥ 12% and 0.2 -L improvement in FEV_1_	425 (48.9)
≥ 10% improvement in PEF	412 (47.4)
≥ 15% improvement in PEF	291 (33.5)
≥ 15% and 60 L/min improvement in PEF	239 (27.5)
≥ 20% improvement in PEF	210 (24.2)
≥ 25% improvement in PEF	149 (17.1)

*Note:* Continuous variables are shown as mean (SD) and categorical variables as *n* (%).

**Table 2:** Characteristics of different PEF increase cut-offs in detecting a 12% and 0.2-L improvement in FEV_1_ during bronchodilation.

**Increase in PEF**	**Sensitivity (%)**	**Specificity (%)**	**PPV (%)**	**NPV (%)**	**Kappa**
10%	75.1	79.1	77.4	76.8	0.541
15%	58.1	90.1	84.9	69.2	0.485
15% and 60 L/min	49.6	93.7	88.3	66.0	0.438
20%	44.7	95.5	90.5	64.3	0.406
25%	32.9	98.0	94.0	60.4	0.313

## Data Availability

The data that support the findings of this study are available on request from the corresponding author. The data are not publicly available due to privacy or ethical restrictions.

## Supporting Information

Supporting Information Additional supporting information can be found online in the Supporting Information section. The characteristics of different PEF cut-off values when BDR is defined as an increase in FEV1 of at least 10% of predicted value, according to the ATS/ERS technical spirometry standard recommendation.
