# Supplementary material for: Relation of Changes in PEF and FEV1 During Salbutamol-Induced Bronchodilation After Methacholine Challenge Test
Source: Pulm Med. 2025 Jul 7;2025:7675935. doi: 10.1155/pm/7675935 (PMC12259322; doi:10.1155/pm/7675935)
Supplement: Supporting Information — Additional supporting information can be found online in the Supporting Information section. The characteristics of different PEF cut-off values when BDR is defined as an increase in FEV1 of at least 10% of predicted value, according to the ATS/ERS technical spirometry standard recommendation. [file 7675935.f1.docx]

# Supplementary information

# The characteristics of different PEF cut-off values when BDR is defined as an increase in FEV_1_ of at least 10% of predicted value, according to the ATS/ERS technical spirometry standard recommendation.

We analyzed the sensitivity, specificity, positive predictive value (PPV), negative predictive value (NPV), and Cohen’s kappa coefficient for different peak expiratory flow (PEF) improvement cut-off values in detecting an increase in forced expiratory volume in 1 second (FEV_1_) at least 10% of predicted value during bronchodilation. We analyzed relative increases in PEF as well as percent predicted increases in PEF. We found that, using this percent predicted definition of bronchodilation, based on kappa value, PEF was even less effective in predicting BDR compared to BDR defined as an increase in FEV_1_ of 12% & 0.2 L in relation to baseline. The varying PEF cut-off values, whether defined as relative increases from baseline or as percent predicted increases, were ranked by accuracy in the same order as when BDR was defined as a 12% & 0.2 L increase in FEV_1_ from baseline. A 10% improvement in both relative and percent predicted PEF increase was found to be the most accurate.

**Table S1.** Characteristics of different cut-off values for relative PEF increase in detecting an improvement in FEV_1_ at least 10% of predicted value after bronchodilation.

| Increase in PEF | Sensitivity (%) | Specificity (%) | PPV (%) | NPV (%) | Kappa |
| --- | --- | --- | --- | --- | --- |
| 10% | 72.5 | 77.3 | 76.0 | 74.0 | 0.498 |
| 15% | 56.3 | 89.0 | 83.5 | 67.3 | 0.454 |
| 15% & 60 L/min | 47.9 | 92.7 | 86.6 | 64.3 | 0.407 |
| 20% | 42.8 | 94.3 | 88.1 | 62.5 | 0.372 |
| 25% | 31.9 | 97.5 | 92.6 | 59.2 | 0.295 |

**Table S2.** Characteristics of different cut-off values for percent predicted PEF increase in detecting an improvement in FEV_1_ at least 10% of predicted value after bronchodilation.

| Increase in PEF | Sensitivity (%) | Specificity (%) | PPV (%) | NPV (%) | Kappa |
| --- | --- | --- | --- | --- | --- |
| 10% of predicted | 64.6 | 84.0 | 79.9 | 70.6 | 0.486 |
| 15% of predicted | 40.3 | 94.3 | 87.4 | 61.5 | 0.347 |
| 20% of predicted | 25.9 | 97.9 | 92.6 | 57.2 | 0.240 |
